# Autologous blood extracellular vesicles and specific CD4^+^ T-cell co-activation

**DOI:** 10.3389/fimmu.2022.992483

**Published:** 2022-09-12

**Authors:** Déborah Neyrinck-Leglantier, Marie Tamagne, Sasha L’honoré, Léonie Cagnet, Sadaf Pakdaman, Alexandre Marchand, France Pirenne, BenoÎt Vingert

**Affiliations:** ^1^ Univ Paris Est-Creteil, INSERM, IMRB, Creteil, France; ^2^ Etablissement Français du Sang, Ivry-sur-Seine, France; ^3^ Laboratory of Excellence GR-Ex, Paris, France; ^4^ Université Paris-Saclay, Laboratoire AntiDopage Français (LADF), Châtenay-Malabry, France

**Keywords:** CD4+ T lymphocytes, sCD27, immunoregulation, autotransfusion, autologous extracellular vesicles

## Abstract

Extracellular vesicles (EVs), which are generated by cell membrane budding in diverse cells, are present in variable numbers in the blood. An immunoregulatory role has been demonstrated principally for heterologous EVs, but the function of the EVs present naturally in blood remains unknown. We hypothesize that these autologous EVs might also modulate the phenotype and function of immune system cells, especially CD4^+^ T lymphocytes (TLs), as previously described for heterologous EVs. Several membranes and soluble immunoregulatory molecules were studied after the treatment of CD4^+^ TLs with autologous EVs. No direct activation was detected with autologous EVs, contrasting with the findings for heterologous EVs. However, following treatment with autologous EVs, a soluble form of CD27 (sCD27) was detected. sCD27 is strongly associated with lymphoproliferation. Autologous EVs have been shown to increase TL proliferation only after T-cell receptor (TcR) engagement due to polyclonal or specific-antigen stimulation. Our results therefore suggest that the EVs present in the blood have an immunomodulatory role different from that of heterologous EVs. These findings should be taken into account in future studies, particularly those focusing on infectious diseases, autotransfusion or doping practices.

## Introduction

There are two types of extracellular vesicle (EVs): exosomes, which are small vesicles (40-100 nm, derived from intracellular membrane compartments), and larger extracellular vesicles (300-900 nm in diameter). The largest extracellular vesicles, also known as microparticles or ectosomes, are generated by the budding of the cell membrane; they play a major role in intercellular communication under physiological and pathological conditions (1). In this study, we focused exclusively on these larger vesicles budding from the plasma membrane of cells, which we will refer to hereafter as EVs. Most studies on EVs have shown an immunoregulatory role for heterologous EVs in transfusion models (2–6). By contrast, the role of the EVs naturally present in blood remains unclear (3). However, these autologous EVs can also be associated with disease. Indeed, the immunoregulatory properties of EVs have been highlighted in autoimmune diseases (1–7).

Blood EVs have diverse cellular origins and their numbers are highly variable; many factor contribute to this variability (1, 2). These variations affect immune system activation, as shown, particularly, for the activation and differentiation of CD4^+^ T lymphocytes (TLs) induced by heterologous EVs (2–5). The variability of autologous EVs may play a similar role under physiological conditions.

We tested this hypothesis, by investigating the functionality of autologous EVs with CD4^+^ TLs. We treated purified CD4^+^ TLs from healthy donors (HDs) with different concentrations of EVs. This dose-effect assay covered the range of variability of the number of EVs observed under physiological conditions (2). We explored the expression of several immunoregulatory molecules on the cell surface (OX40/OX40L, CD40/CD40L, PD1 and ICOS) and in the culture supernatant: CD357 (GITR), CD270 (HVEM), IDO, CD30, CD40, DR6, CD95 (Fas), CD120a/b (TNF-R1 and -R2), TRAIL-R1/-R2, CD272 (BTLA), CD223 (LAG-3), PD1/PD-ligands, CD366 (Tim-3), CD28/CD80, CD137 (4-1BB), CD27 and CD152 (CTLA-4). All these molecules were selected on the basis of major roles they play in immune system regulation or their possible association with EVs (1, 2, 8–12). We also investigated whether autologous EVs had the same lymphoproliferative properties as heterologous EVs in response to polyclonal or specific-antigen stimulation (2, 3).

## Materials and methods

### Biological samples

Fresh blood samples from healthy blood donors (HDs) were collected for the isolation of EVs and cells. Samples were provided by the French national blood bank (*Etablissement Français du Sang*, EFS). None of the HDs had had an infection (bacterial, viral, fungal, yeast) or had been vaccinated in the 30 days preceding inclusion, and all gave written informed consent.

### CD4^+^ TL purification

PBMCs were isolated from fresh blood samples by density gradient centrifugation. CD4^+^ TLs were purified from PBMCs by negative selection with a commercial kit (IMag CD4 TLymphocyte Enrichment kit, BD Biosciences, NJ, Franklin Lakes). Following cell enrichment, CD4^+^ TLs were sorted by flow cytometry (Aria Fusion, BD Biosciences), to achieve a purity greater than 99%. Live/Dead Fixable Aqua cell staining kits were used to exclude dead cells (Thermo Fisher Scientific, MA, Waltham).

### EV isolation and phenotyping

EVs were isolated from fresh blood samples by differential centrifugation at an initial speed of 3,000 x *g* at 4°C for 10 minutes. The supernatant was then centrifuged at 13,000 x *g* at 4°C for the preparation of a platelet-free supernatant. EV phenotyping was performed on this platelet-free supernatant. EVs were labeled as previously described, with fluorochrome-conjugated monoclonal antibodies (2, 13). All EVs were labeled with the following antibodies: anti-CD3 BV510, anti-CD4 BV711, anti-CD8 BUV737, anti-CD11c BUV395, anti-CD14 PE-Cy7, anti-CD19 AF700, anti-CD41a APC-H7, anti-CD142 PE and anti-CD235a PE-Cy5 (BD Biosciences), anti-CD16 BV605 and anti-CD123 BV421 (Biolegend, San Diego, CA) antibodies. EVs were acquired at low speed (200 events/s) and were quantified in Trucount tubes (BD Biosciences). Fluorescence was assessed with a 20-parameter LSR Fortessa flow cytometer with a small-particle option (BD Biosciences), in this case, photomultiplier (PMT)-coupled forward scatter (FSC) detection. This mode of detection was used to ensure the optimal detection of EVs with diameters of 300 to 900 nm. The performance of the flow cytometer was checked before each assay. Megamix-Plus FSC polystyrene beads (BioCytex, Marseille, France) of known dimensions (300 nm; 500 nm and 900 nm beads, mean diameter) were used to standardize PMT- FSC parameters and to define the EV gate.

For CD4^+^ TL treatments, EVs were concentrated by centrifuging the platelet-free supernatant for 1 hour at 100,000 x *g* and 4°C and resuspending the pellet in filtered culture medium (filter with 0.1 µm pores). The culture medium consisted of RPMI 1640 supplemented with 5% FBS (Dutscher, Bernolsheim, France), 2 mM L-glutamine, 100 µg/ml penicillin/streptomycin, MEM Non-Essential Amino Acids Solution (1X), and 1 mM sodium pyruvate (all from Life Technologies, Carlsbad, CA).

### CD4^+^ TL activation assay

We cultured 1 x 10^4^ sorted CD4 T cells in filtered culture medium (filter with 0.1 µm pores) as previously described (2, 13), for six days with autologous EVs at ratios (CD4^+^ TLs: EVs) of 50:1, 20:1, 1:1 and 1:20. The supernatant was removed and frozen at -80°C for the assessment of cytokine secretion by the Luminex method. Cells were then labeled with the following antibodies for analysis by LSR Fortessa flow cytometry: anti-CD134/OX40 BV421, anti-CD252/OX40L PE, anti-CD40 PE-Cy7, anti-CD154/CD40L PE-CF594, anti-CD279/PD1 PerCP-Cy5.5, and anti-CD278/ICOS BV711 (BD Biosciences) antibodies.

### Soluble immune checkpoint expression

We assessed the levels of 22 soluble immune checkpoints in the supernatant of CD4^+^ TL: EV cultures, in a ProcartaPlex simplex assay performed in accordance with the manufacturer’s instructions (Thermo Fisher Scientific). Bead fluorescence was read with a MAGPIX reader (Luminex, Austin, TX). For matrix visualization analysis, a heat map was generated with Morpheus software (The Eli and Edythe Broad Institute of MIT and Harvard Cambridge, MA).

### CD4^+^ TL proliferation assay

We assessed CD4^+^ T-cell proliferation, as previously described (2). We stained 1 x 10^4^ purified CD4^+^ TLs with CFSE and cultured them in filtered culture medium (filter with 0.1 µm pores) with autologous EVs at ratios (CD4^+^ TLs: EVs) of 1:1 and 1:20 in the absence (NS) or presence of antigen stimulation (staphylococcal enterotoxin B (SEB) or tuberculin-purified protein derivative (PPD), 1 µg/mL) for six days. Cell division was then assessed by analyzing CD4^+^CFSE^lo^ TL levels with a Fortessa flow cytometer (BD Biosciences). Lymphoproliferation was normalized between donors. For each HD tested, a proliferation index of 1 was assigned to the lymphoproliferation observed in the absence of EVs. Lymphoproliferation is expressed proportionally, as the fold-induction relative to lymphoproliferation in the absence of EVs.

### CD27^+^ EV sorting

EVs were labeled with anti-CD27 BV510 antibody (Biolegend) for sorting with a MoFlo Astrios flow cytometer (Beckman Coulter, Brea CA) equipped with a PMT-FSC detector, as previously described (2). Flow cytometer performance was assessed before the sorting experiments. Polystyrene beads (BioCytex) of known dimensions (300 nm, 500 nm and 900 nm in diameter) were used to standardize PMT-FSC parameters and to define the total MP gate.

### Flow cytometry analysis

Flow cytometry data were analyzed with FlowJo software (v.10.7.1, Ashland, OR).

### Statistical analysis

All analyses were performed with Prism 6.07 software (GraphPad Software, La Jolla, CA). The significance of differences was determined in Kruskal-Wallis tests and Dunn *post-hoc* tests. In all figures, only statistically significant differences between groups (*P*<0.05) are indicated.

## Results

### Direct plasma EV phenotyping

Direct plasma EV phenotyping was performed to characterize the EVs from 22 HDs. Cellular origin was assessed for red blood cell EVs (RBC EVs), platelet EVs (PEVs), monocyte EVs (MEVs), dendritic cell EVs (DC EVs), CD16 EVs, CD4 EVs, CD8 EVs, endothelial EVs (EEVs) and B-lymphocyte EVs (LB EVs) (**Supplemental Figure 1**). The numbers and size distribution of these EVs were also assessed in relation to their cellular origin (**Supplemental Table 1**). This direct plasma EV phenotyping revealed heterogeneity in the cellular origin of EVs (**Table 1**). Three types of EVs predominated (each with a frequency of more than 10%) — RBC EVs, PEVs and MEVs — together accounting for 80.1% of the EVs present in plasma studied.

**Table 1:** Cellular origin of EVs from the plasma of 22 HDs.

		Mean ± SD (x106)*	% EVs
RBC EVs	(CD235a^+^)	4.87 ± 9.90	42.8
PEVs	(CD41a^+^)	2.92 ± 6.70	25.6
MEVs	(CD14^+^)	1.33 ± 4.99	11.7
DC EVs	(CD123^+^ CDl c^+^)	0.81 ± 0.47	7.1
CD16 EVs	(CD16^+^)	0.55 ± 0.52	4.9
CD4 EVs	(CD4^+^)	0.33 ± 0.19	2.9
CD8 EVs	(CD8^+^)	0.27 ± 0.20	2.4
Endo EVs	(CD142^+^)	0.15 ± 0.10	1.3
LB EVs	(CD19^+^)	0.14 ± 0.09	1.2

*Number of EVs/mL of plasma*

### Autologous EVs and CD4^+^ TL membrane activation profile

**Figure 1:**
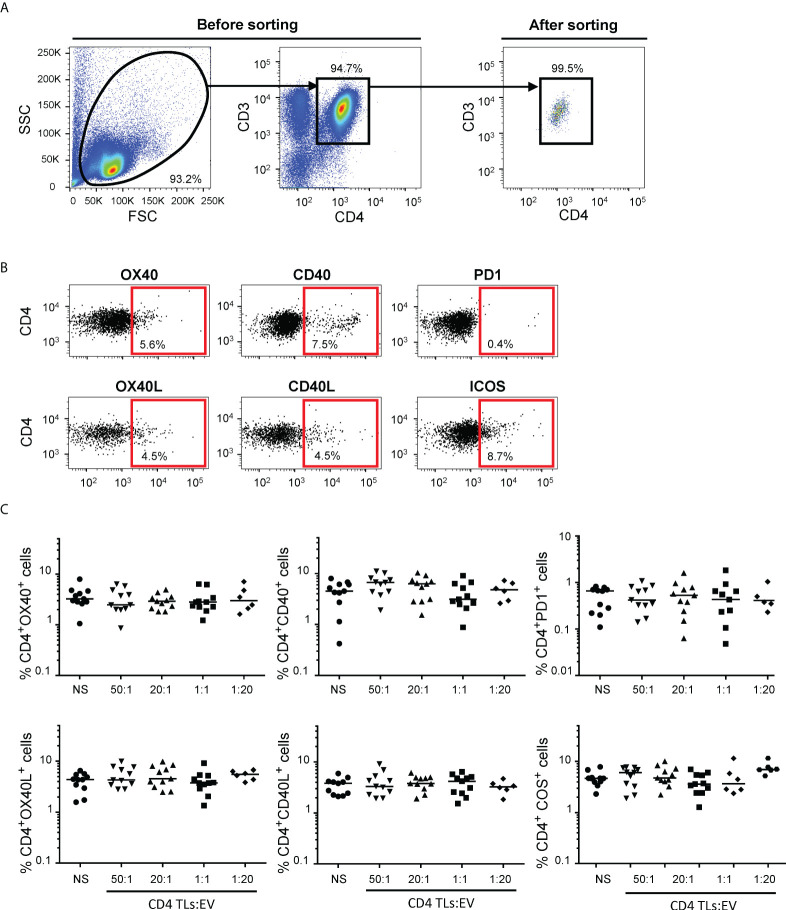
Flow cytometry analysis of CD4^+^ TL activation for cultures of sorted CD4^+^ TLs and autologous EVs. **(A)** Gating strategy for the sorting of CD4^+^ T cells. Dot plots for total enriched CD4^+^ TLs before (on the left) and after (on the right) sorting by flow cytometry. **(B)** Representative FACS plots from 11 experiments measuring the expression of OX40, OX40L, CD40, CD40L, PD1 and ICOS on CD4^+^ TLs. **(C)** OX40, OX40L, CD40, CD40L, PD1 and ICOS expression on the surface of CD4^+^ TLs cells was assessed after incubation without EVs (○) or treatment with total autologous EVs at ratios (CD4: EVs) of 50:1 (▾), 20:1 (▴). 1:1 (▪) (from 11 HDs) and 1:20 (♦) (from 6 HDs) for six days. Horizontal bars indicate the median.

The immunoregulatory functions of autologous blood EVs were investigated with purified CD4^+^ TLs (**Figure 1A**). Autologous CD4^+^ TLs were treated with EVs in different ratios to reproduce the variability of EV numbers (**Supplemental Figure 1**, **Supplemental Table 1**). We then studied the expression of six membrane receptors: OX40, CD40, PD1, OX40L, CD40L and ICOS (**Figure 1B**). Regardless of the number of EVs, we observed no change in the membrane activation profile of CD4^+^ TLs after 48 hours (data not shown) or six days of culture (**Figure 1C**).

### Autologous EVs and secretion of immunoregulatory molecules by CD4^+^ TLs

**Figure 2:**
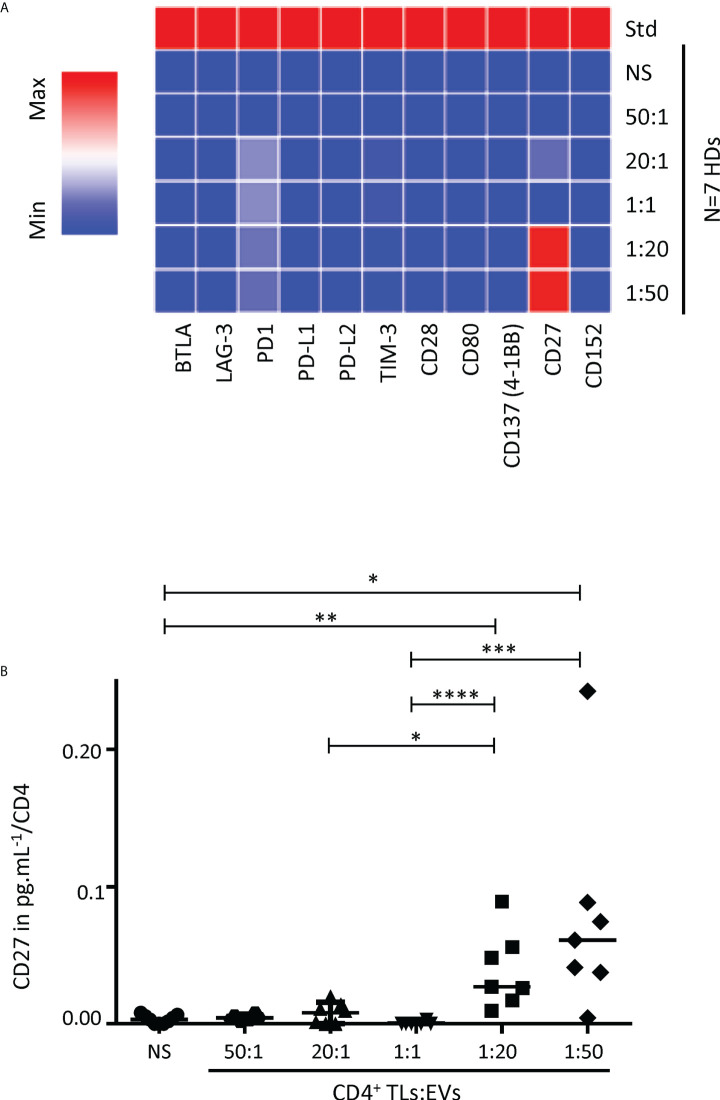
Analysis of soluble immune checkpoint levels in the supernatant of cultures of sorted CD4^+^ TLs and autologous EVs. **(A)** The levels of 11 soluble immune checkpoints were determined with Luminex technology. A heatmap was generated to compare soluble immune checkpoint concentrations between culture supernatants from CD4^+^ TLs without EVs (NS) or treated with total autologous EVs at different ratios (CD4: EVs). **(B)** sCD27 levels in supernatants collected after six days of CD4: EV culture, assessed in pg/mL per CD4^+^ TL for incubation without EVs (NS, ○), or treatment with EVs at different ratios (CD4: EVs): 50:1 (**○**), 20:1 (▴), 1:1 (▾), 1:20 (▪) and 1:50 (♦) for six days (from 7 HDs). Std indicates the standard values for the soluble immune checkpoint. Horizontal bars indicate the median. *p* values for comparisons were obtained in Kruskal-Wallis tests and Dunn *post-hoc* tests: *p<0.05, **p<0.01, ***p<0.001, ****p<0.0001.

We also studied the immunoregulatory effects of autologous blood EVs on purified CD4^+^ TLs, by exploring 22 soluble immunoregulatory molecules (human TNF receptor super family and immune checkpoint) produced by CD4^+^ TLs. None of the 22 molecules studied was produced by CD4^+^ TLs after 48 hours of culture, regardless of the numbers of autologous EV in culture (data not shown). None of the human TNF receptor super family studied was produced by CD4^+^ TLs after six days of culture, regardless of the numbers of autologous EV in culture (data not shown). However, after six days of treatment, soluble CD27 (sCD27) was detected in significant amounts, with a dose-dependent effect of EVs (**Figure 2A**). Significant sCD27 secretion was detected for EV ratios of 1:20 and 1:50 (*P*<0.01, *P*<0.05, CD4^+^ TLs: EVs), with 3.9x10^-2^ ± 1.1x10^-2^ pg/ml and 7.8x10^-2^ ± 7.7x10^-2^ pg/ml per CD4^+^ TL cultured, respectively, versus 0.4 x 10^-2^ ± 0.1 x 10^-2^ pg/ml per CD4^+^ TL cultured for the control (**Figure 2B**).

### Autologous EVs and CD4^+^ TL proliferation

**Figure 3:**
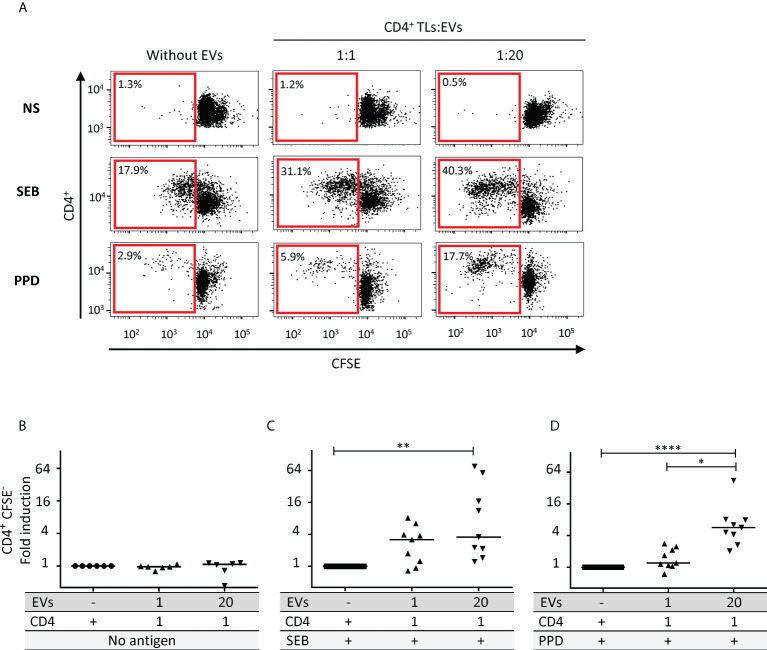
Effect of autologous EVs on CD4^+^ TL lymphoproliferation. **(A)** Sorted CD4 T cell from HDs were labeled with CFSE and cultured without EVs or with autologous EVs at ratios (CD4: EVs) of 1:1 (▴) and 1:20 (▾) for six days in the absence (NS) or presence of antigen (SEB or PPD) at 1 µg/mL. Cell division was assessed after six days of stimulation, by timed acquisition flow cytometry analysis for CD4^+^CFSE^lo^ T cells. Results from one representative sample are shown. **(B)** CD4^+^ TL lymphoproliferation was assessed for six HDs, for sorted CD4^+^ TLs cultured without EVs (○) or with total autologous EVs at ratios (CD4: EVs) of 20:1 (♦), 1:1 (▴) and 1:20 (▾) for six days. **(C)** CD4^+^ TL lymphoproliferation was assessed for nine HDs, for preparations enriched in CD4^+^ TLs stimulated with SEB and cultured without EVs (▪) or with total autologous EVs at ratios (CD4: EVs) of 20:1 (♦), 1:1 (▴) and 1:20 (▾) for six days. **(D)** CD4^+^ TL lymphoproliferation was assessed for nine HDs, for PBMCs stimulated with PPD and cultured without EVs (▪) or with total autologous EVs at ratios (CD4: EVs) of 1:1 (▴) and 1:20 (▾) for six days. Horizontal bars indicate the median. *p* values for comparisons were obtained in Kruskal-Wallis tests and Dunn *post-hoc* tests: *p<0.05, **p<0.01, ****p<0.0001.

We investigated the effect of increasing numbers of autologous EVs on CD4^+^ TL proliferation, as previously described for heterologous EVs (2). We treated autologous CD4^+^ TLs with EVs at two different CD4^+^ TL: EV ratios: 1:1 and 1:20 (**Figure 3A**). Regardless of the number of EVs, no lymphoproliferation was observed (**Figure 3A, B**). However, following SEB superantigen stimulation, we observed a significant increase in lymphoproliferation in the presence of EVs at a ratio of 1:20 (*P*<0.01, **Figure 3A, C**). Proliferation rates were 19.1 ± 9.3 times higher in these conditions than in the absence of EVs. The role of these autologous EVs in TcR engagement was also investigated with CD4^+^ TLs from healthy vaccinated donors with positive tuberculin skin test (PPD) results. Once again, lymphoproliferation rates were significantly higher in the presence of EVs at a treatment ratio of 1:20 (*P*<0.001, **Figure 3A, D**). Proliferation rates in these conditions were 9.5 ± 4.4 times higher than those in the absence of EVs.

## Discussion

We mimicked the conditions in the bloodstream *in vivo* as closely as possible, by studying the autologous blood EVs present in plasma directly, without ultracentrifugation. The results obtained with this direct phenotyping method were similar to published results, particularly for the phenotyping of EVs in HDs (2, 3, 14).. This direct plasma EV phenotyping also revealed heterogeneity in the numbers and size distribution of EVs (**Supplemental Table 1**).

Our objective here was to determine whether autologous EVs play a direct role in regulating the immune system, similar to that played by heterologous EVs. The data obtained in this *in vitro* study suggest that, regardless of treatment time, autologous EVs have no direct effect on CD4^+^ TL activation.

However, EVs are involved in intercellular communication (3–5). The absence of variation in the expression of the major immunoregulatory molecules studied suggested a possible indirect action of EVs on autologous CD4^+^ TLs (**Figure 1**). The detection of sCD27 is also consistent with such an indirect effect. sCD27 is an alternative form of the CD27 transmembrane receptor. sCD27 has been detected in the biological fluids of HDs and patients with various diseases (12, 15–18). Conflicting findings have been published concerning sCD27, but most studies have reported that this protein contributes to cell activation and lymphoproliferation following the triggering of the TcR/CD3 complex (12, 19–22). For CD4^+^ TLs, sCD27, in association with its CD70 ligand, has been shown to upregulate the expression of CD40L and to stimulate lymphoproliferation (12, 20, 23). However, no change in CD40L levels was detected in this study (**Figure 1**). Even though this phenotypic change was not observed with these autologous EVs, CD40/CD40L was reported to play a role for a certain type of EV in a previous study (3). Nevertheless, we still do not know whether CD40/CD40L plays an important role in the situation studied here, these molecules being present on EVs or CD4^+^ TLs (2).

However, sCD27 may also be provided by CD27^+^ EVs during culture. Indeed, the presence of CD27^+^ EVs has recently been reported in the plasma of HIV-infected patients (14). We purified CD27^+^ EVs by flow cytometry to test this hypothesis. CD27^+^ EVs were tested with the same Luminex assays used to detect sCD27. We detected no purified CD27^+^ EVs in Luminex assays (**Supplemental Figure 2B**). The sCD27 detected in supernatants cannot, therefore, have originated from the CD27^+^ EVs initially added to the culture.

The reason for the induction of sCD27 by CD4^+^ TLs in response to EVs remains unclear, but it may have consequences for lymphoproliferation. Nevertheless, we followed up on the sCD27 lead and the work of Huang J. *et al.*, by investigating whether sCD27 could promote the lymphoproliferation of stimulated T cells (12). We exposed T cells to polyclonal activation with SEB or antigen-specific PPD restimulation. Once the TcR was committed in response to such stimulation, EVs significantly increased the proliferation of T cells (**Figure 3**). Our data with SEB confirm previously reported data obtained by polyclonal PHA activation (3). However, this previous study did not take the dose effect into account (3), potentially accounting for the lack of a proliferative effect in the absence of mitogen treatment (3). Nevertheless, our results for the PPD vaccinal antigen confirm that autologous EVs facilitate the lymphoproliferation of specific CD4^+^ TLs.

Our data provide evidence of a mechanism of interaction between autologous EVs and CD4^+^ TLs different from that reported for heterologous EVs supplied by transfusion (2). Indeed, unlike heterologous EVs (2), autologous EVs cannot induce conventional CD4^+^ TL proliferation without prior involvement of the TcR. However, Treg, but also circulating Tfh, Th17 or other subpopulations of CD4^+^ TLs play major roles in inducing or controlling adaptive immune responses, and heterologous EVs have been shown to be functional on these cells (2, 4–6). Finally, we investigated whether autologous EVs could have a functional effect on autologous cells at higher concentrations in the blood, by performing functional lymphoproliferation tests on Tfh, Treg and Th17 cells. However, as with conventional CD4^+^ TLs, autologous EVs did not induce the proliferation of these subpopulations (**Supplemental Figure 3**).

Heterologous EVs have been shown to induce lymphoproliferation at low concentrations of between 20,000:1 and 200:1 (2). Studies were performed with additional ratios (200 and 2000:1 with 200,000 cells in culture), but no lymphoproliferation was detected with autologous EVs at any of these ratios (data not shown).

The cellular origin of EVs might underlie functional modifications of the immune system. Several groups have reported functional differences between EVs of different cellular origins in heterologous transfusion studies (3–6). However, these studies did not specifically purify these EVs. EVs from apheresis-derived platelet concentrates or from red blood cell concentrates do not consist exclusively of platelet-EVs or erythrocyte-EVs (2, 3). Nevertheless, studies of EVs from stored blood concentrates have provided an indication as to the potential role of some EVs (3). Indeed, during blood concentrate storage, a change occurs in the source of the EVs present, and a particular type of EVs, such as erythrocyte-EVs, may come to predominate (3).

Moreover, we cannot exclude the possibility that specific autologous EVs with costimulatory molecules are associated with lymphoproliferation after TCR engagement, as already reported for heterologous EVs (2).

**Figure 4:**
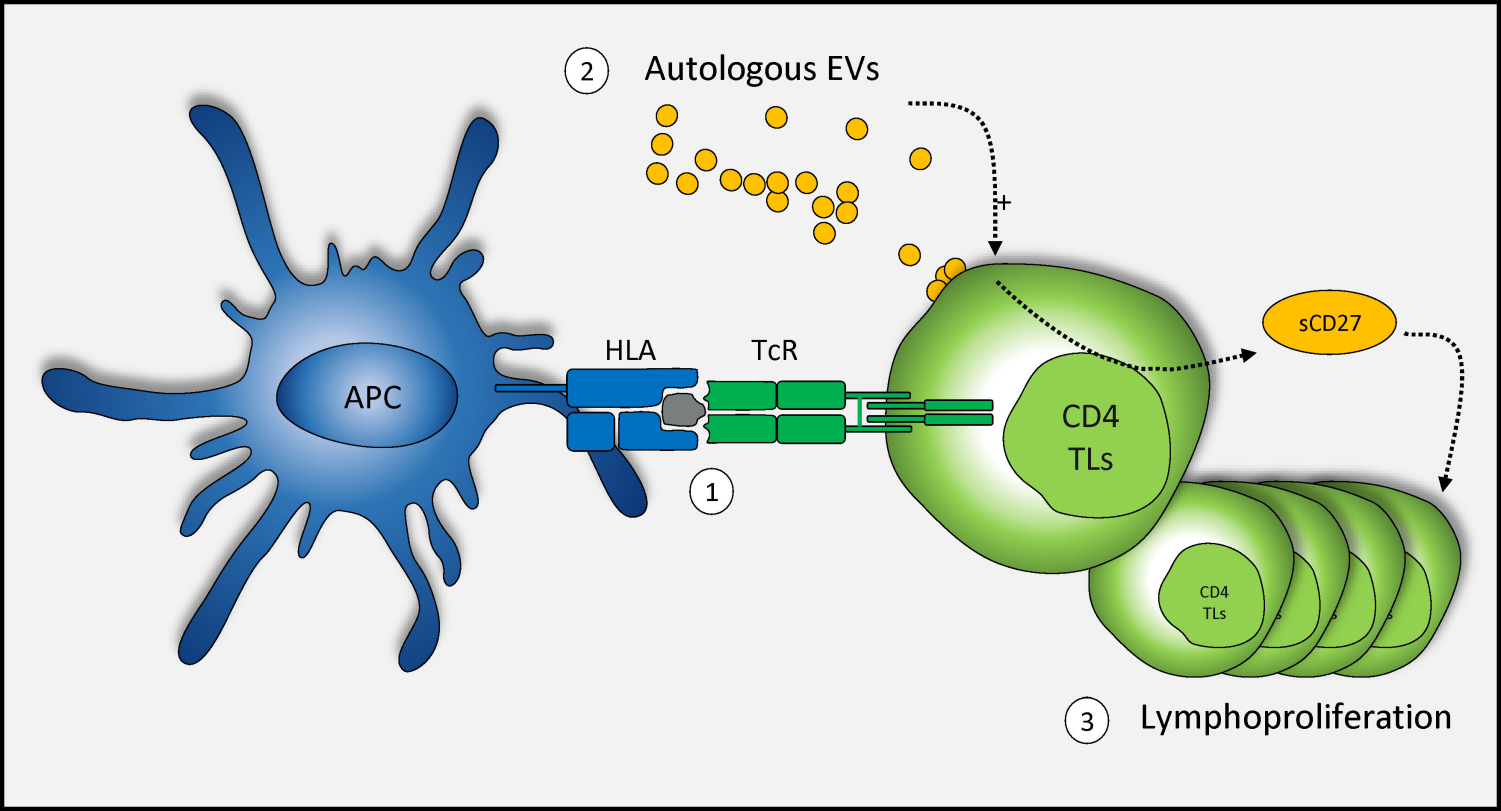
Schematic representation of the mechanism of action of autologous blood EVs in CD4^+^ TL activation. TcR engagement and autologous EVs enhance the T cell-mediated immune response. (1) Once the TcR is committed in response to stimulation, (2) autologous EVs play a role in the secretion of sCD27 (3) potentially leading to a large increase in T-cell proliferation.

EVs naturally present in the blood may play a functional role in intercellular communication in the immune system, by promoting lymphoproliferation after TcR engagement, probably with the assistance of sCD27 (**Figure 4**). However, further studies are required to elucidate the link between sCD27 secretion by autologous CD4^+^ TLs and EVs.

Finally, these autologous EVs may, therefore, play a key role in pathological conditions, particularly in patients in which EV levels are high due to infection, autoimmune disease, or cancer (1, 24–27). It will also be important to take these findings into account in cases of autotransfusion for pathological conditions or in the case of doping practices (28, 29).

## Data availability statement

The original contributions presented in the study are included in the article/**Supplementary Material**. Further inquiries can be directed to the corresponding author.

## Ethics statement

Ethical review and approval was not required for the study on human participants in accordance with the local legislation and institutional requirements. The patients/participants provided their written informed consent to participate in this study.

## Author contributions

BV was the principal investigator and takes primary responsibility for the paper; SP was the clinical research associate for this study. DN-L, SL and MT performed the laboratory work. DN-L and BV analyzed the results; BV and FP coordinated the research; DN-L and BV wrote the paper; FP, LC and AM reviewed the paper. All the authors contributed to the manuscript and approved the submitted version.

## Acknowledgments

This work was supported by the scientific committee of *Agence Française de Lutte contre le Dopage* (AFLD), the World Antidoping Agency (WADA), *Etablissement Français du Sang*, INSERM and *Université Paris-Est Créteil*. We are particularly grateful to the healthy blood donors who participated in this study, and the EFS team responsible for collecting blood donations. We would like to thank Muriel Andrieu and Souganya Many from the flow cytometry facility of the Cochin Institute (CYBIO Core facility).

## Conflict of interest

The authors declare that the research was conducted in the absence of any commercial or financial relationships that could be construed as a potential conflict of interest.

## Publisher’s note

All claims expressed in this article are solely those of the authors and do not necessarily represent those of their affiliated organizations, or those of the publisher, the editors and the reviewers. Any product that may be evaluated in this article, or claim that may be made by its manufacturer, is not guaranteed or endorsed by the publisher.

## Abbreviations

EVs, Extracellular vesicles; RBC EVs, red blood cell EVs; P EVs, platelet EVs; MEVs, monocyte EVs; DC EVs, dendritic cell EVs; EEVs, endothelial EVs; LB EVs, B-lymphocyte EVs; sCD27, soluble form of CD27; SEB, staphylococcal enterotoxin B; PPD, tuberculin-purified protein derivative.
