# Supplementary material for: Autologous blood extracellular vesicles and specific CD4+ T-cell co-activation
Source: Front Immunol. 2022 Sep 12;13:992483. doi: 10.3389/fimmu.2022.992483 (PMC9510993; doi:10.3389/fimmu.2022.992483)
Supplement: Supplementary file 1 [file DataSheet_1.docx]

**Autologous blood extracellular vesicles and specific CD4^+^ T-cell co-activation**

Déborah Neyrinck-Leglantier, Marie Tamagne, Sasha L’honoré, Léonie Cagnet, Sadaf Pakdaman, Alexandre Marchand, France Pirenne and Benoît Vingert

**Supplemental Figures and tables**

**
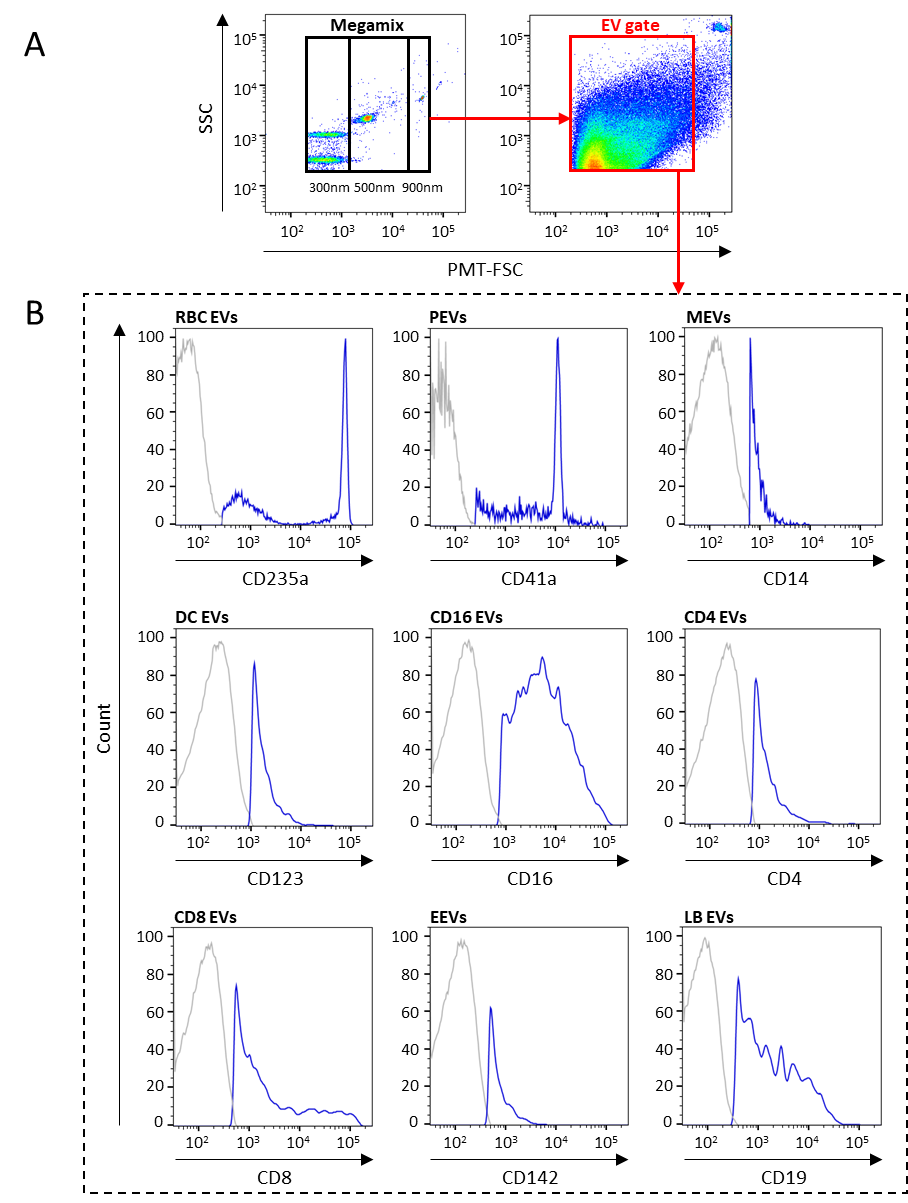
**

**Supplemental Figure 1: Gating strategy for the phenotyping of EVs from HD blood.** (A) The phenotype of EVs was investigated by flow cytometry on plasma samples from 22 HDs. On the left, a dot plot showing the settings based on fluorescent beads for the differentiation of three particle sizes: 300, 500 and 900 nm in diameter. On the right, dot plot for EV acquisition on a Fortessa flow cytometer for one representative HD. (B) RBC EVs, PEVs, MEVs, DC EVs, CD16 EVs, CD4 EVs, CD8 EVs, EEVs and LB EVs were gated in the EV gate. Representative normalized FACS histogram drawn by FlowJo from HDs. In this FlowJo representation, the positive peak (in blue) is normalized to the height of the negative peak (in grey) allowing a better visualization of the molecule labeling aspect on the EVs.

**
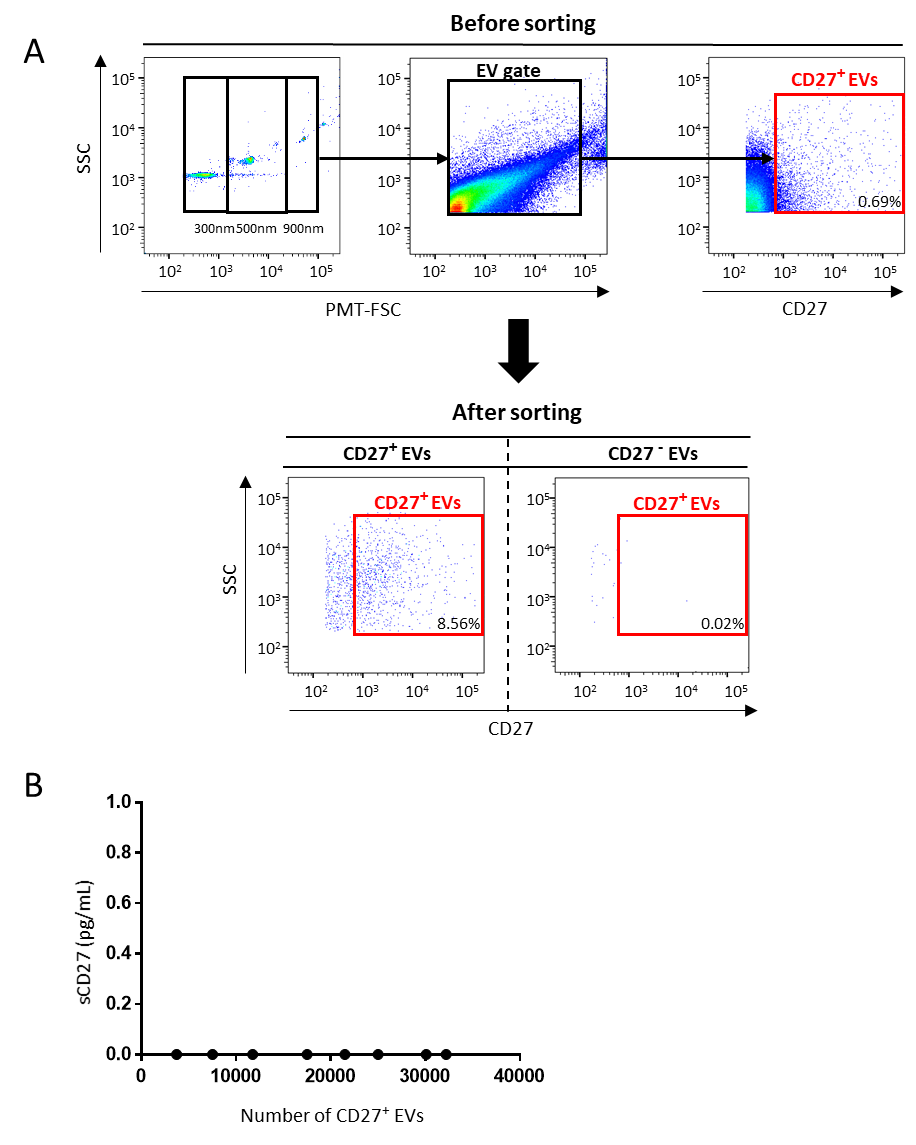
**

**Supplemental Figure 2: Detection of CD27 production by EVs.** (A) Dot plots showing the gating strategy for CD27^+^ EVs before (top) and after (bottom) sorting by flow cytometry. The performance of the MoFlo Astrios flow cytometer was checked with Megamix beads before cell sorting. (B) Correlation between CD27^+^ EVs and sCD27 levels.


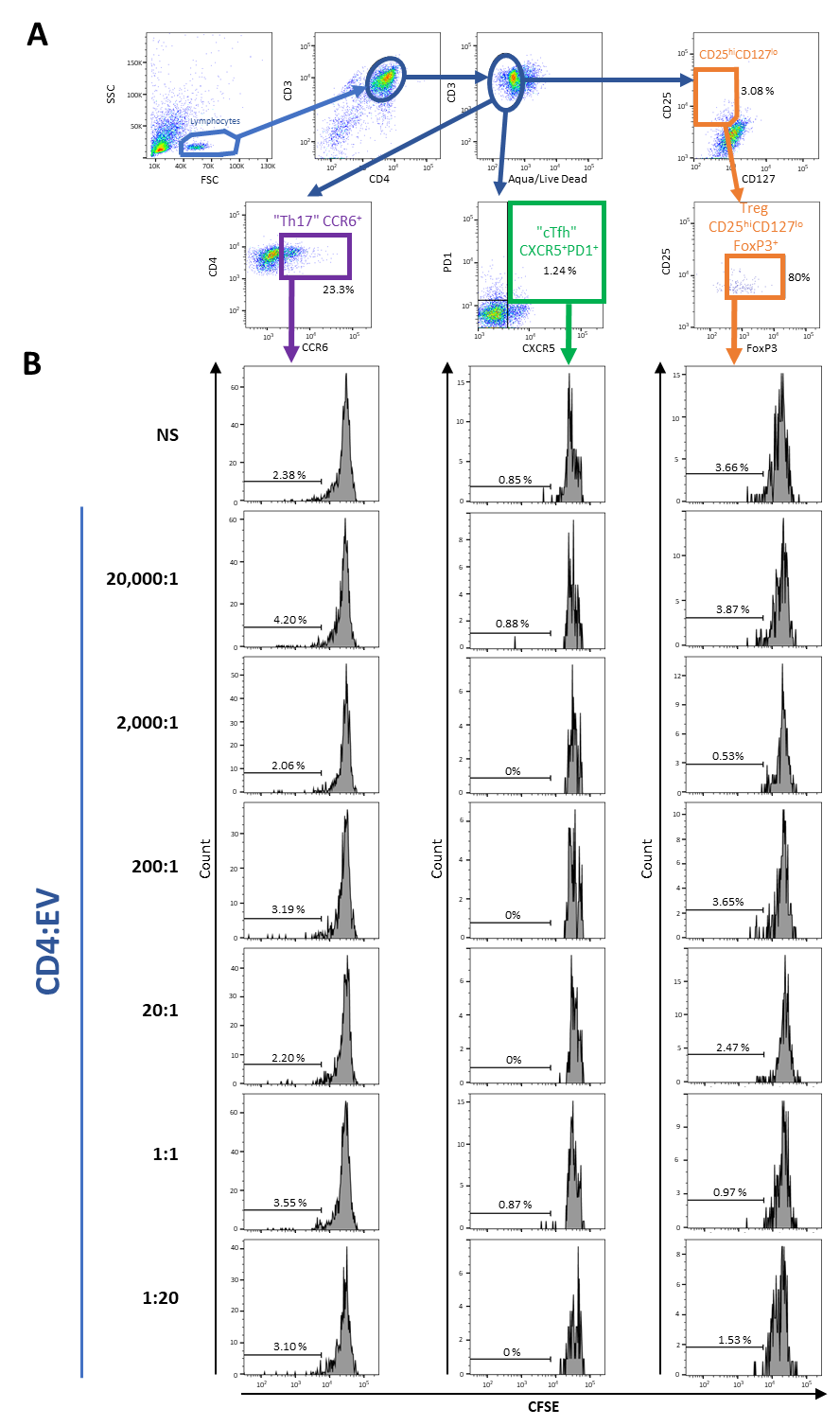


**Supplemental Figure 3: Lymphoproliferation analysis after autologous EVs treatment on circulating Th17 cells (CCR6^+^), circulating Tfh cells (CXCR5^+^PD1^+^) and Tregs (CD127^lo^CD25^hi^FoxP3^+^) from CD4^+^ TLs.** (A) Gating strategy of Th17, cTfh and Tregs. (B) PBMCs from three HDs were treated with autologous EVs for 6 days, and lymphoproliferation was assessed by flow cytometry. Example of histograms showing the proliferation of Th17, Tregs and Tfh cells with and without autologous EVs. Cell division was assessed with the CFSE^lo^ LT subpopulation (data are from representative of three experiments with one donor per experiment).

**Supplemental Table 1: Number of total EVs in plasma from the blood of 22 HDs**
